# Epigenetic control of the angiotensin-converting enzyme in endothelial cells during inflammation

**DOI:** 10.1371/journal.pone.0216218

**Published:** 2019-05-01

**Authors:** Thomas Mudersbach, Daniel Siuda, Karin Kohlstedt, Ingrid Fleming

**Affiliations:** 1 Institute for Vascular Signalling, Centre for Molecular Medicine, Goethe University, Frankfurt am Main, Germany; 2 German Centre for Cardiovascular Research (DZHK), Partner site Rhein-Main, Frankfurt am Main, Germany; University of Bonn, Institute of Experimental Hematology and Transfusion Medicine, GERMANY

## Abstract

The angiotensin-converting enzyme (ACE) plays a central role in the renin-angiotensin system, which is involved in the regulation of blood pressure. Alterations in ACE expression or activity are associated with various pathological phenotypes, particularly cardiovascular diseases. In human endothelial cells, ACE was shown to be negatively regulated by tumor necrosis factor (TNF) α. To examine, whether or not, epigenetic factors were involved in ACE expression regulation, methylated DNA immunoprecipitation and RNA interference experiments directed against regulators of DNA methylation homeostasis i.e., DNA methyltransferases (DNMTs) and ten-eleven translocation methylcytosine dioxygenases (TETs), were performed. TNFα stimulation enhanced DNA methylation in two distinct regions within the ACE promoter via a mechanism linked to DNMT3a and DNMT3b, but not to DNMT1. At the same time, TET1 protein expression was downregulated. In addition, DNA methylation decreased the binding affinity of the transcription factor MYC associated factor X to the ACE promoter. In conclusion, DNA methylation determines the TNFα-dependent regulation of ACE gene transcription and thus protein expression in human endothelial cells.

## Introduction

The angiotensin-converting enzyme (ACE or CD143) is an ectoenzyme that plays a central role in the generation of the angiotensin II as well as in the degradation of bradykinin, and thus has consequences on the regulation of blood pressure [1] and vascular remodeling [2]. Alterations in endothelial ACE expression or activity are associated with inflammatory cardiovascular diseases, including diabetes [3] and atherosclerosis [4]. The consequences of ACE inhibition should not be attributed solely to its ability to cleave angiotensin I and bradykinin as ACE can regulate inflammation through mechanisms independent of angiotensin II synthesis and kinin breakdown. For example, the N–and C–catalytic domains of the ACE enzyme are also able to process substrates such as the anti-inflammatory tetrapeptide N-acetyl–seryl-asparyl-lysyl-proline [5]. Given these considerations it is rather intriguing that while ACE inhibitors are used to effectively treat vascular disease, endothelial cell activation in response to the inflammatory cytokine tumor necrosis factor (TNF) α, decreases rather than increases ACE expression [6].

ACE expression has been reported to increase in vascular smooth muscle cells following exposure to fluid shear stress [7] or pulsatile pressure changes [8]. The transcription factor Krüppel-like factor 2, which is activated by shear stress [9], has also been linked to ACE expression in endothelial cells as overexpression of the transcription factor decreases levels of the protein [10]. More recently, the consequences of shear stress on ACE expression were also linked to a mechanism involving the AMP–activated protein kinase, the phosphorylation of p53 and upregulation of miR-143/145 [11]. There is growing evidence that epigenetic alterations are involved in the pathological mechanisms of many chronic disorders. As a general rule, hypermethylation of a gene promoter CpG region tends to silence gene expression, while the CpG island hypomethylation results in the opposite response. Importantly, the human *ACE* gene promoter has been shown to harbor CpG islands [12], and CpG island hypomethylation of the ACE gene has been linked with fetal programming and the potential development of later disease [13]. Surprisingly little is known about how inflammatory stimuli can influence cellular ACE activity. Given that inflammation leads to global DNA hypermethylation and hence persistent changes in gene expression [14,15], the aim of this study was to assess the role of DNA methylation in the TNFα-dependent regulation of ACE expression in endothelial cells.

## Materials and methods

### Cell culture

Human umbilical endothelial cells were isolated and purified from veins and arteries of umbilical cords using VE-cadherin (CD144) antibody-coated magnetic beads from Dynal Biotech (Brown Deer/Wisconsin, USA) as described [16] (dx.doi.org/10.17504/protocols.io.ybdfsi6). Endothelial cells were cultured in MCDB131 medium from Gibco (Invitrogen, Karlsruhe, Germany) supplemented with 10 mmol/L L-glutamine, 8% fetal calf serum, 1 g/L NaHCO_3_, 50 mg/L penicillin, 50 mg/L streptomycin, 0.1 μg/L epidermal growth factor, 1 μg/L fibroblast growth factor and 2 mL/500 mL endothelial cell growth supplement plus heparin from Promocell (Heidelberg, Germany, #C-30120). For all experiments, cells were used between passages 1 and 4. The use of human material in this study conforms to the principles outlined in the Declaration of Helsinki [17], and the isolation of endothelial cells was approved in written form by the ethic committee of the Goethe University.

### Cell treatment

Human endothelial cells were treated with TNFα (PeproTech, Hamburg, Germany, #300-01A) for the times indicated in the results section. Preliminary concentration response experiments revealed 10 ng/mL TNFα as an optimal concentration with a maximum effect on ACE expression and low cell toxicity. For wash out experiments, cells were washed twice with basal MCDB131 medium and further cultured in normal growth medium (without TNFα). The TNFα receptor was inhibited by pretreatment with R-7050 (10 μmol/L) from Santa Cruz (Dallas/Texas, USA, CAS#303997-35-5) 30 minutes prior to stimulation. For the RNA stability assay, cells were pretreated with the RNA polymerase II inhibitor 5,6-dichloro-1-β-D-ribofuranosylbenzimidazole (DRB; 20 μg/mL; Cayman Chemical, Ann Arbor/Michigan, USA, CAS#53-85-0) two hours prior to stimulation. RG-108 (30 μmol/L; Active Motif, La Hulpe, Belgium, #14104) was used to inhibit DNMT. The p38 mitogen activated protein kinase inhibitor; SB202190 was from Calbiochem (Darmstadt, Germany).

### Small interfering RNA (siRNA)

Cells were transfected at approximately 80% of confluence. siRNAs against DNMT1 (Ambion ID:110914; Thermo Fisher Scientific, Massachusetts, USA), DNMT3a (Ambion ID:s200426), DNMT3b (Ambion ID:111746) and TET1 (Ambion ID:147894) were used at a final concentration of 50 μmol/L. Transfection was carried out using Lipofectamine RNAiMAX from Invitrogen (Karlsruhe, Germany) according to the manufacturer’s instructions (dx.doi.org/10.17504/protocols.io.xtqfnmw).

### Chromatin immunoprecipitation (ChIP)

DNA was extracted using the DNeasy Blood Mini Kit from Qiagen (Hilden, Germany). DNA was sheared in ice-cold water (20/20 rounds high/low shear of 10 seconds on/off cycles) using the Bioruptor plus from Diagenode (Seraing, Belgium) to achieve fragments of 200-1000 bp in length. ChIP assay was performed using a chromatin immunoprecipitation kit (Merck Millipore #17–10085; Darmstadt, Germany) according to the manufacturer’s instructions. The MYC associated factor X (MAX) antibody used to detect MAX-binding to DNA was from Abcam (Cambridge, UK). Changes in the DNA methylation status were measured by using a methylated DNA immunoprecipitation kit (Diagenode, Seraing, Belgium,). Immunoprecipitated DNA was analyzed by qPCR. ACE specific primers were designed to flank the MAX binding sites upstream of the transcription start site (from -1336 bp to -1135 bp; “upstream”) and within the CpG island (from +27 bp to +318 bp; “CpG”) as listed in Table 1. All samples were normalized to their corresponding input and solvent treated cells.

**Table 1:** Primer sequences for qPCR.

Primer		Sequences
ChIP_ACE-upstream	forward	5‘-CGGCCAGTGTTTAACAAGGC-3‘
	reverse	5‘-GCTCCAAGTCACCTCAGGTC-3‘
ChIP_ACE-CpG	forward	5‘-GCCTGCAAGACACCTAAGGTC-3‘
	reverse	5‘-TTCCTCCTCCGCTCCAGAG-3‘
ChIP_ACE-6kb up control	forward	5‘-GATCACACCACTGCACTCCA-3‘
	reverse	5‘-GGAAACTAGGGCTGCTTCGT-3‘
18S	forward	5‘-CTTTGGTCGCTCGCTCCTC-3‘
	reverse	5‘-CTGACCGGGTTGGTTTTGAT-3‘
ACE	forward	5‘-AATTGCCTTCCTGCCCTTTG-3‘
	reverse	5‘-TACCACCAGTCGAAGTTGTAGC-3‘
COX-2	forward	5‘-GCCCGACTCCCTTGGGTGTC-3‘
	reverse	5‘-CTGATGCGTGAAGTGCTGGGCA-3‘
DNMT1	forward	5‘-TGCCAGCTGAGCGTGGTGGT-3‘
	reverse	5‘-GCATGCGGGCAGCCACCAAT-3‘
DNMT3a	forward	5‘-CCGGAACATTGAGGACATCT-3‘
	reverse	5‘-CAGCAGATGGTGCAGTAGGA-3‘
DNMT3b	forward	5‘-GCAAAGACCGAGGGGATGAA-3‘
	reverse	5‘-CTGCCACAAGACAAACAGCC-3‘
GAPDH	forward	5‘-ATGACATCAAGAAGGTGGTG-3‘
	reverse	5‘-CATACCAGGAAATGAGCTTG-3‘
IL-6	forward	5‘-GCCTTCGGTCCAGTTGCCTT-3‘
	reverse	5‘-GCAGAATGAGATGAGTTGTC-3‘
MAX	forward	5‘-TCAGCAAAATGGTCCTGTGG-3‘
	reverse	5‘-AAGGTTGTAAGGCCAGAGTCAG-3‘
TATA-box binding protein	forward	5‘-CACGAACCACGGCACTGATT-3‘
	reverse	5‘-TTTTCTTGCTGCCAGTCTGGAC-3‘
TET1	forward	5‘-TCTGTTGTTGTGCCTCTGGA-3‘
	reverse	5‘-CCCATGACCACATCTACTGT-3‘
TET2	forward	5‘-AGCAATAGGACATCCCTGAG-3‘
	reverse	5‘-CATCTAGGAGCAGGTCCTAA-3‘
TET3	forward	5‘-CGGATCGAGAAGGTCATCTA-3‘
	reverse	5‘-ATGACGATCACAGCGTTCTG-3‘

### Real-time quantitative PCR (RT-qPCR)

Total RNA was isolated using peqGOLD TriFast (VWR, Darmstadt, Germany) and 300 ng were reversely transcribed using SuperScript III reverse transcriptase (Life Technologies, Darmstadt, Germany). Real-time qPCR from equal amounts of cDNA was performed using Magnetic Induction Cycler from Biozym (Hessisch Oldendorf, Germany) and a SYBR Green master mix (Thermo Fisher Scientific, Massachusetts, USA). The relative expression levels of the different genes studied were calculated using the ^ΔΔ^Ct method and normalized to the geometric mean of the housekeeping genes (HKG) glyceraldehyde-3-phosphate dehydrogenase, TATA-box binding protein and 18S ribosomal RNA in each sample. The primers used (Table 1) were from Biospring (Frankfurt, Germany).

### Immunoblotting

Cells were lysed in Nonidet lysis buffer: 20 mmol/L Tris (pH 8.0), 1% Nonidet, 137 mmol/L NaCl, 25 mmol/L β-glycerophosphate, 2 mmol/L Na_4_P_2_O_7_, 10% glycerol, freshly enriched with protease and phosphatase inhibitors. Equal amounts of protein were separated on SDS-PAGE and transferred to nitrocellulose membranes. After blocking for one hour in Roti-block from Carl Roth (Karlsruhe, Germany), membranes were incubated in primary antibody (4°C overnight), followed by detection using secondary antibody (1:20.000) coupled to horseradish peroxidase. Proteins were visualized by enhanced chemiluminescence.

### Antibodies

For Western blotting all antibodies were diluted in Roti-block. The anti-ACE antibody (1:1000) was a kind gift from Dr. Peter Bünning (Aventis, Frankfurt). Anti-non-muscle myosin heavy chain (NMM) (ab75590, 1:2000; Cambridge, UK), anti-MAX (ab53570, 1:1000) and anti-DNMT1 (ab13537, 1:1000) were purchased from Abcam (Cambridge, UK), anti-TET1 (sc-293186, 1:500) was from Santa Cruz (Heidelberg, Germany), and the β-actin antibody (A5541, 1:5000) was from Sigma (Darmstadt, Germany).

### Statistics

Unless otherwise indicated values are expressed as the mean ± SEM, and statistical evaluation was performed using Student’s *t* test for analysis between two groups containing normally distributed data and one-way ANOVA followed by the Newman-Keuls post-test or two-way ANOVA followed by the Bonferroni post-test for comparisons between multiple groups. All statistical calculations were performed using Prism 7 software (GraphPad Inc., La Jolla, California). Values of P<0.05 were considered statistically significant.

## Results

### Effect of TNFα on ACE expression in human endothelial cells

**Fig 1:**
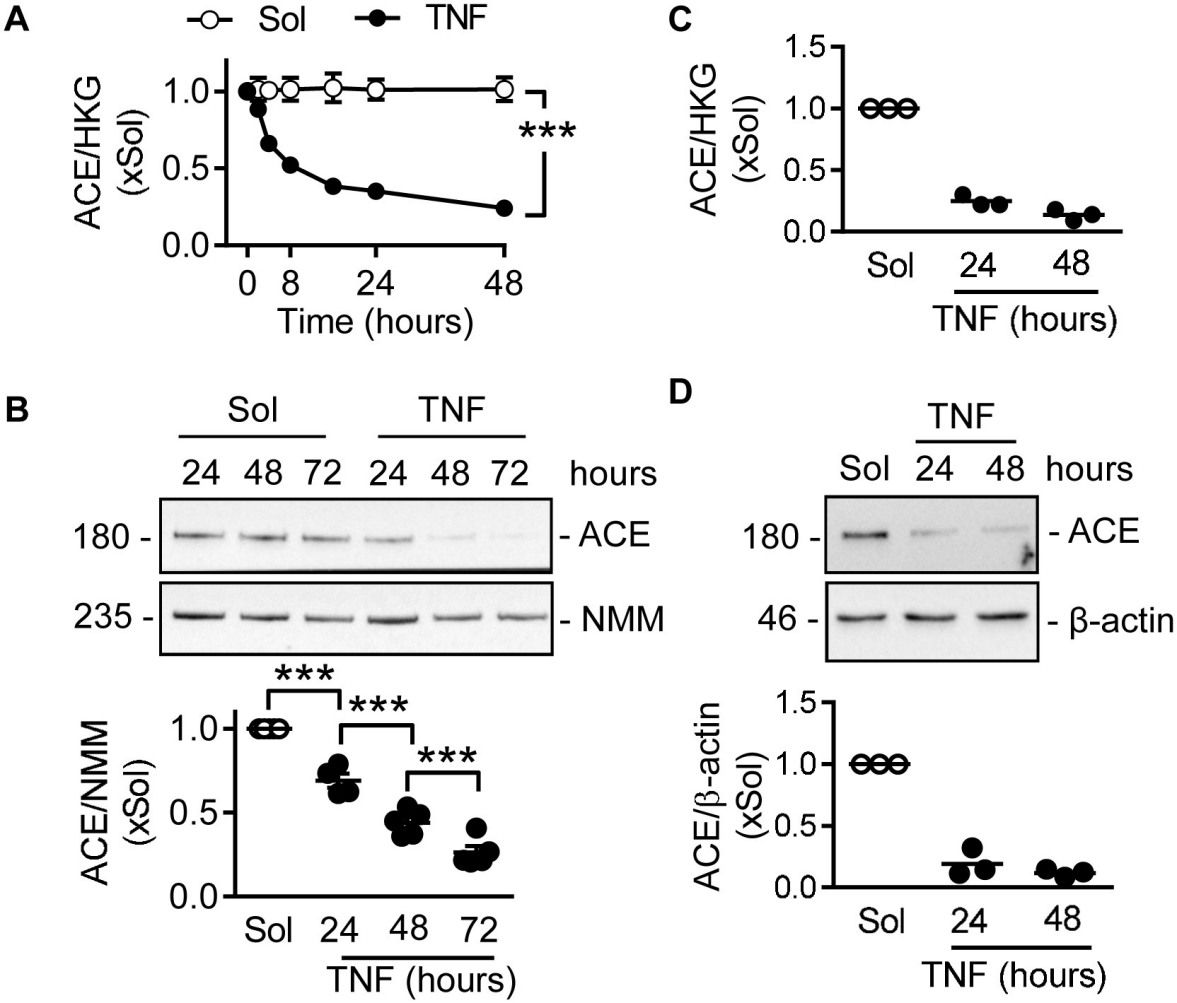
Effect of TNFα on ACE mRNA and protein expression in human endothelial cells. (A) Time course of the effects of TNFα (10 ng/mL) on ACE mRNA levels in human umbilical vein endothelial cells relative to a triplet of housekeeping genes (HKG); n = 6 independent cell batches (two-way ANOVA/Bonferroni). (B) Time course of the effects of TNFα on ACE protein expression in human umbilical vein endothelial cells relative to non-muscle myosin heavy chain (NMM); n = 4–5 independent cell batches (one-way ANOVA/Newman-Keuls). (C&D) Expression of ACE mRNA (C) and protein (D) in human umbilical artery endothelial cells; n = 3 independent cell batches. ***P<0.001.

The treatment of human umbilical vein endothelial cells with TNFα for up to 72 hours resulted in the significant downregulation of ACE mRNA (Fig 1A) and protein (Fig 1B) levels. The effect was not restricted to venous endothelial cells as similar results were obtained using human umbilical artery endothelial cells (Fig 1C&1D). The observations could be directly attributed to TNFα, rather than an indirect mechanism as the TNFα receptor antagonist R-7050 abrogated the cytokine-induced downregulation of ACE mRNA (S1A Fig).

To determine whether or not TNFα destabilized ACE mRNA, cells were treated with TNFα in the absence and presence of the RNA-polymerase II inhibitor DRB. The turnover of ACE mRNA was relatively fast as eight hours after DRB treatment ACE mRNA levels were decreased by 32.7 ± 9.2% (S1B Fig). Stimulation with TNFα in addition to DRB had, however, no additional effect on ACE mRNA levels (decrease of 35.7 ± 7.4%). Given that TNFα was previously reported to attenuate ACE expression by a mechanism involving p38 mitogen-activated protein kinase (MAPK) [6], studies were performed using the p38 MAPK inhibitor, SB202190. However, p38 MAPK inhibition was without effect on the TNFα-induced downregulation of ACE in primary endothelial cells (S1C Fig), indicating that alternative mechanisms play a more dominant role in its regulation.

### TNFα-induced changes at the ACE promoter region

**Fig 2:**
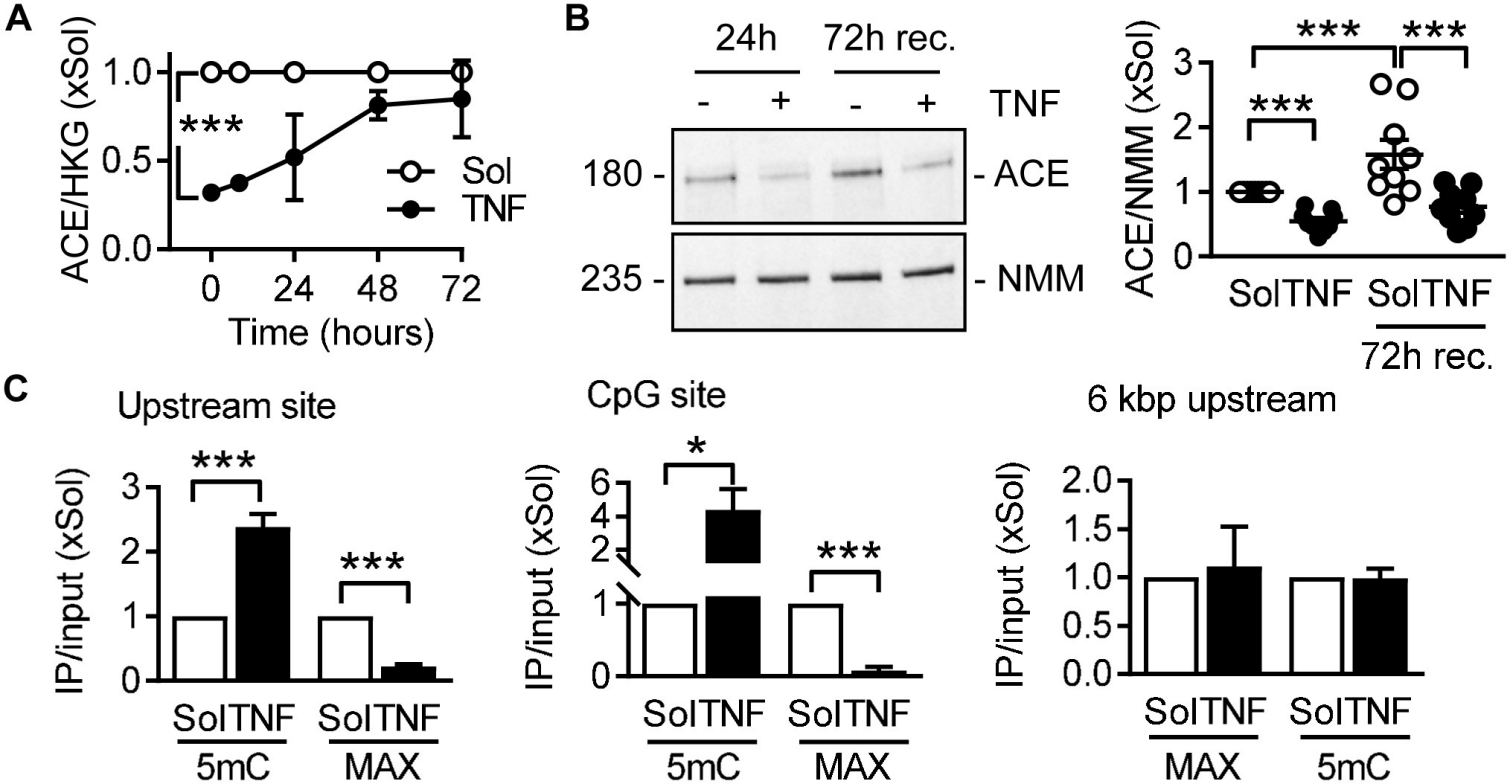
Involvement of epigenetic factors in TNFα-mediated changes in ACE expression. (A&B) Recovery (rec.) of ACE mRNA (A) and protein (B) in human endothelial cells after 24 hours TNFα (10 ng/mL) stimulation followed by washout for up to 72 hours; n = 6, relative to a triplet of housekeeping genes (HKG) (two-way ANOVA/Bonferroni, A) and n = 9, relative to non-muscle myosin heavy chain (NMM) (one-way ANOVA/Newman-Keuls, B). (C) Effect of TNFα (10 ng/mL, 24 hours) on DNA methylation and MAX binding to three distinct sites within the ACE promoter, quantified by chromatin immunoprecipitation (IP), n = 5–11 independent cell batches (Student’s *t*-test). *P<0.05, ***P<0.001.

To assess the recovery of ACE expression, endothelial cells were incubated with TNFα for 24 hours. Thereafter, the cells were washed and ACE expression was monitored for up to 72 hours. This procedure resulted in a slow recovery of ACE mRNA (Fig 2A) but not protein expression (Fig 2B). This delay in recovery was suggestive of regulation by epigenetic mechanisms.

The ACE promoter region (http://genome.ucsc.edu, GRCh37/hg19; ACE: NM_000789; S2A Fig) contains a CpG island, that spans from -612 base pairs (bp) to +605 bp, relative to the transcription start site (TSS), containing 139 CpG-sites, as well as two binding sites for the transcription factor MYC associated factor X (MAX). Chromatin immunoprecipitation (ChIP) experiments were performed to investigate TNFα-induced changes in the DNA methylation status of ACE and the binding of the transcription factor MAX, using appropriate antibodies. The primer pairs used were designed to bind to the precipitated DNA, covering the two MAX binding sites “upstream site” (from -1336 bp to -1135 bp upstream of the TSS) and the “CpG site” (+27 bp to +318 bp) within the ACE promoter region. Another set of primers covered the region from -6008 bp to -5888 bp relative to the TSS of the ACE gene and served as an internal control. TNFα stimulation increased cytosine methylation within the ACE promoter region at the upstream site, as well as in the CpG site, while the internal control was unaffected (Fig 2C). These changes in DNA methylation were associated with a decrease in MAX binding without any effect on global MAX expression (S2B Fig). These observations suggest that the methylation of the CpG islands in the ACE promoter modify ACE expression by directly affecting MAX binding.

### Role of DNMTs in TNFα-mediated ACE expression

**Fig 3:**
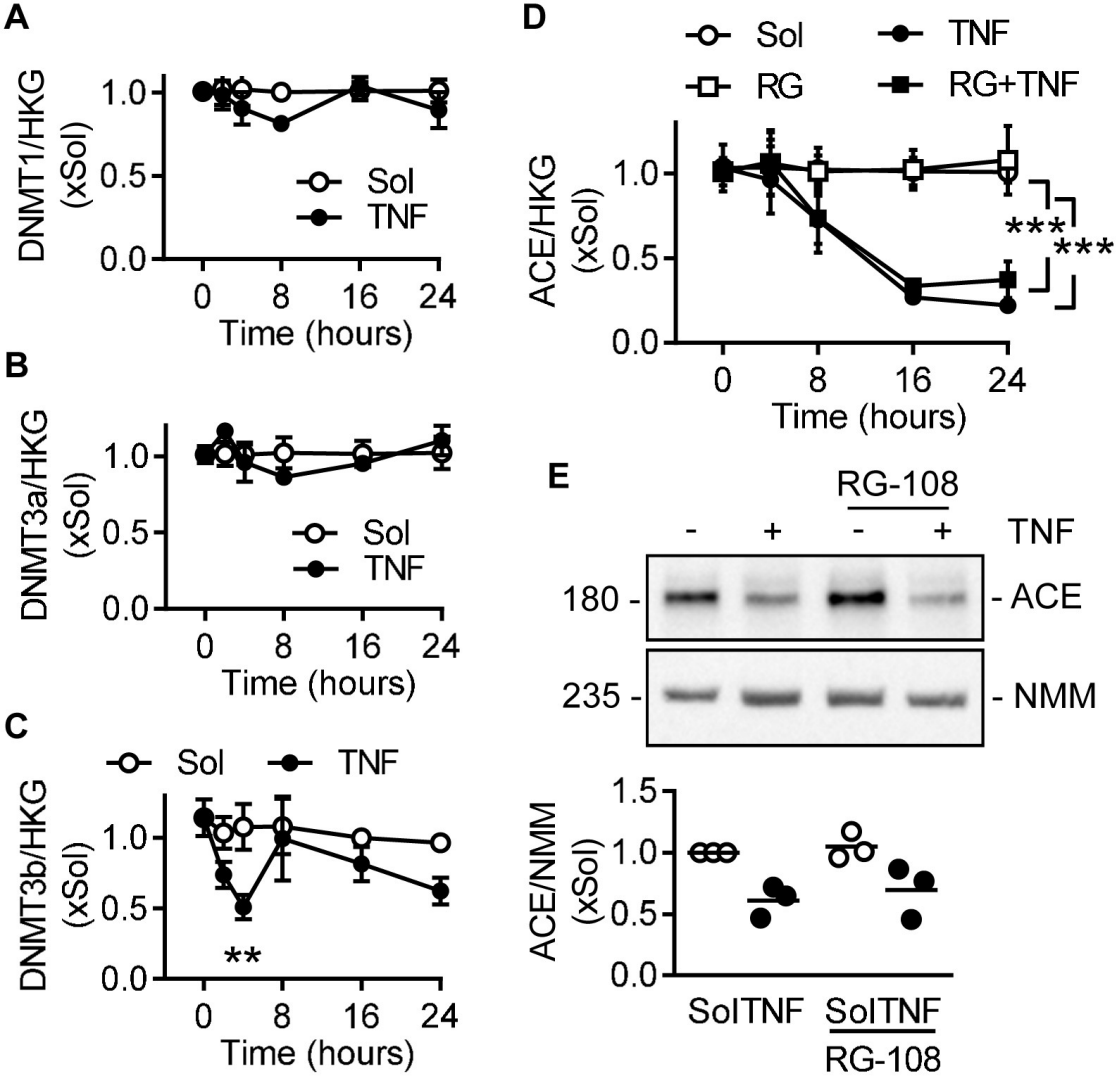
Expression of DNMTs in TNFα-treated human endothelial cells. (A-C) Time course of the effects of TNFα (10 ng/mL) on DNMT1 (A), DNMT3a (B) and DNMT3b (C) mRNA levels in human endothelial cells relative to a triplet of housekeeping genes (HKG); all n = 6 independent cell batches (two-way ANOVA/Bonferroni). (D-E) Effect of RG-108 (RG, 30 μmol/L) on the expression of ACE in human endothelial cells treated with solvent (Sol) or TNFα (10 ng/mL). Time course of ACE mRNA (D); n = 4 independent cell batches (two-way ANOVA/Bonferroni) and ACE protein (E) relative to non-muscle myosin heavy chain (NMM) 24 hours after treatment; n = 3 independent cell batches. **P<0.01, ***P<0.001.

**Fig 4:**
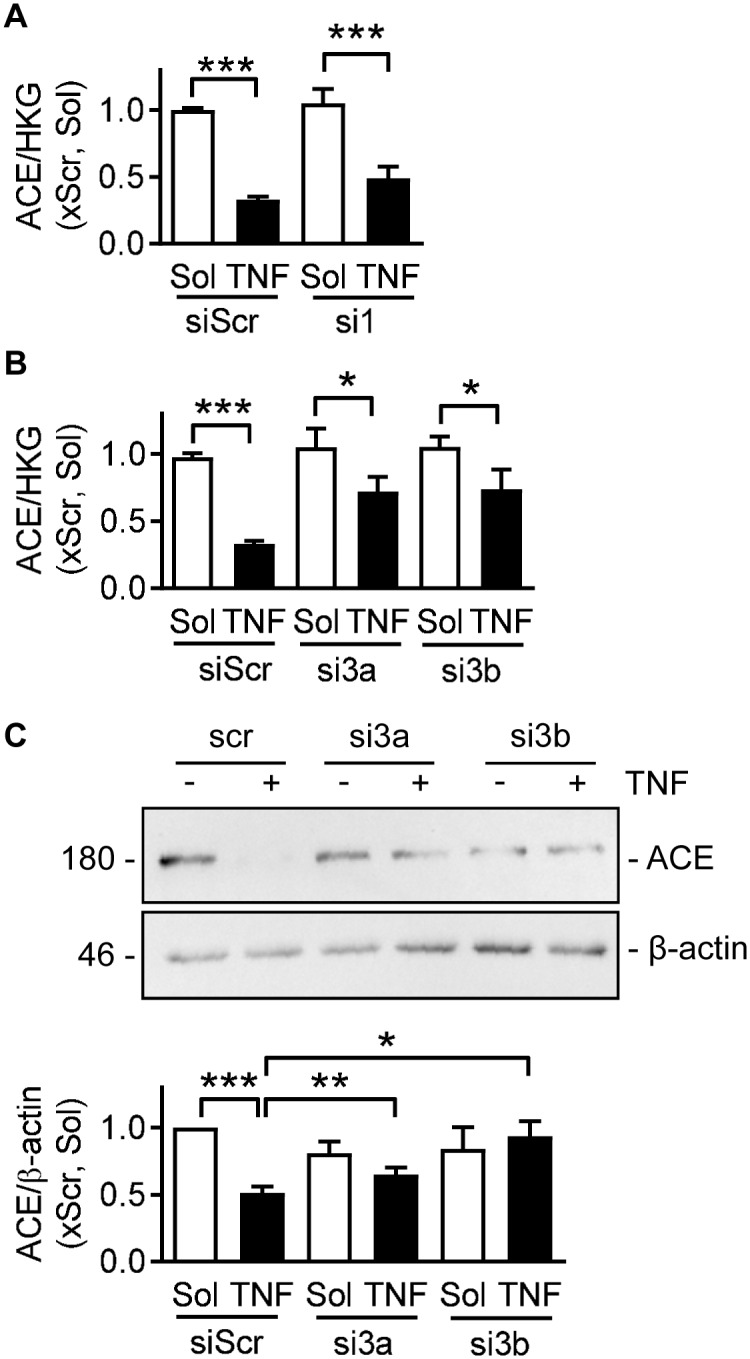
Involvement of DNMTs in TNFα-mediated alterations in ACE expression in human endothelial cells. (A) Consequences of DNMT1 downregulation (si1) on solvent (Sol) and TNFα (10 ng/mL, 24 hours)-induced changes in ACE mRNA relative to a triplet of housekeeping genes (HKG). A scrambled (Scr) oligonucleotide was used as control, n = 4–6 independent cell batches (two-way ANOVA/Bonferroni). (B&C) Consequences of DNMT3a (si3a) and 3b (si3b) downregulation on solvent (Sol) and TNFα (10 ng/mL, 24 hours)-induced changes in ACE mRNA (B) and protein (C) expression. A scrambled (Scr) oligonucleotide was used as control, n = 5–9 independent cell batches (two-way ANOVA/Bonferroni). *P<0.05, **P<0.01, ***P<0.001.

Since TNFα induced the DNA methylation of the ACE promoter, the consequences of TNFα on the expression of DNA methyltransferases (DNMT) were assessed. TNFα failed to alter the expression of DNMT1 (Fig 3A) or DNMT3a (Fig 3B), but induced a transient decrease in DNMT3b after four hours (Fig 3C). The pan-DNMT inhibitor; RG-108 (30 μmol/L), did not alter either the expression of ACE mRNA (Fig 3D) or protein (Fig 3E). However, RG-108 was designed to bind to the catalytic domain of DNMT1 and was only predicted to bind to the other two isoforms [18,19]. Therefore, to investigate the role of DNMT proteins in more detail the different family members (DNMT1, DNMT3a and DNMT3b) were downregulated using a siRNA approach (S3 Fig). While the downregulation of DNMT1 did not affect the TNFα-induced decrease in ACE expression (Fig 4A), the response was largely prevented by the downregulation of DNMT3a or DNMT3b (Fig 4B&4C). Thus, TNFα-induced changes in ACE expression are at least partly regulated by DNMT3a and DNMT3b.

### Involvement of TET enzymes in TNFα-dependent ACE expression

The process of DNA methylation is catalyzed by DNMT enzymes [20] but no single enzyme is able to directly remove the methyl group from the 5’-methylcytosine (5mC) [21]. Rather, demethylation is achieved via several intermediate steps and members of ten-eleven translocation methylcytosine dioxygenase (TET) family of proteins oxidize 5mC to 5’-hydroxymethylcytosine (5hmC) [22,23], which is genomically stable and opposes the actions of 5mC [24].

**Fig 5:**
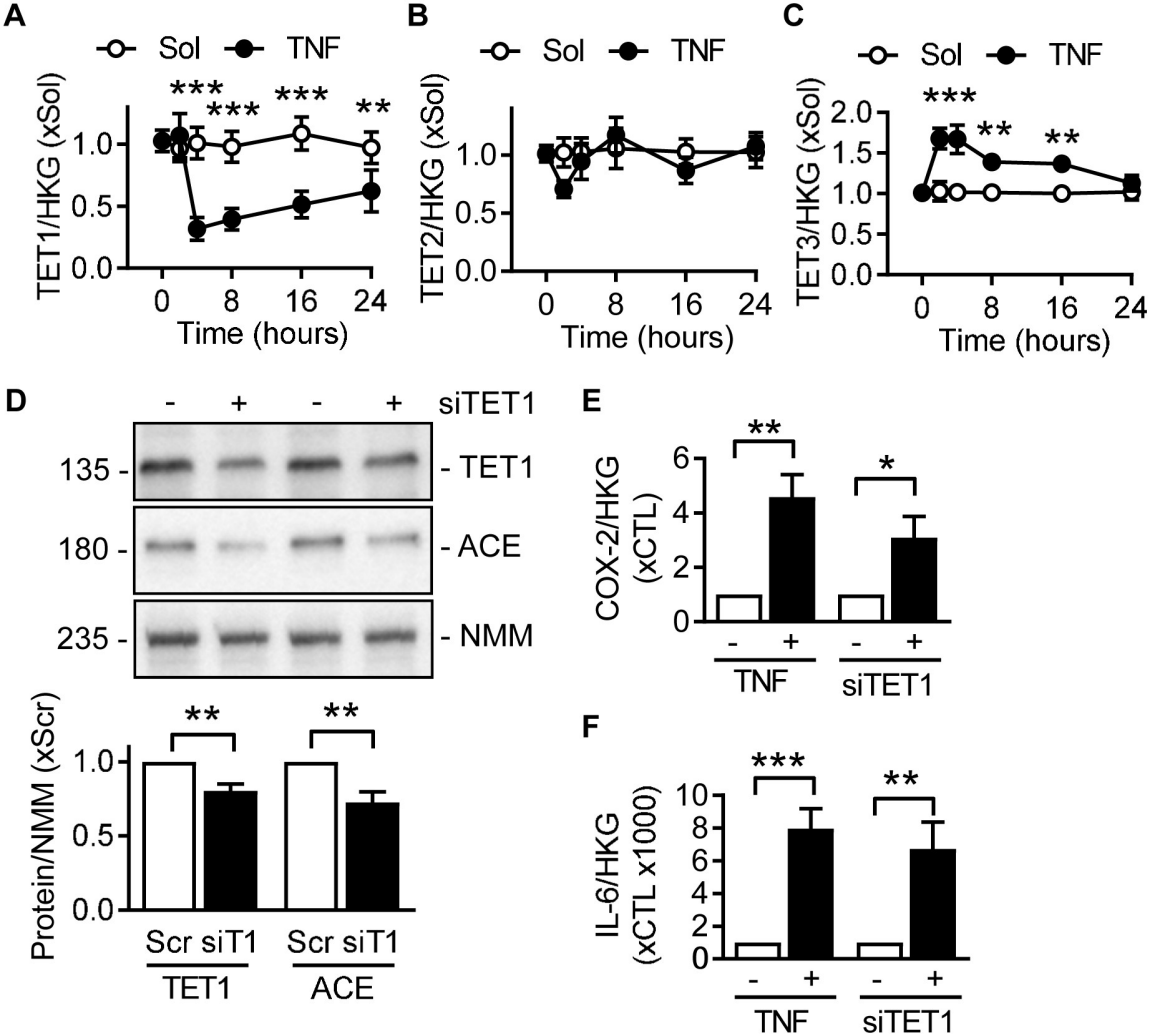
TET1-dependency of TNFα-mediated ACE downregulation in human endothelial cells. (A-C) Time course of the effects of TNFα (10 ng/mL) on TET1 (A), TET2 (B) and TET3 (C) mRNA levels in human endothelial cells relative to a triplet of housekeeping genes (HKG); all n = 5–6 independent cell batches (two-way ANOVA/Bonferroni). (D) RNA interference experiments (50 μmol/L) against TET1 (siT1). A scrambled (Scr) oligonucleotide was used as control. Protein expression levels of TET1 (left panel) and ACE (right panel) versus non-muscle myosin heavy chain (NMM); n = 4-5 independent cell batches (Student’s *t*-test). (E-F) Effect of TNFα (10 ng/mL, 24 hours, left panel) and siRNA against TET1 (50 μmol/L, right panel) on the expression of cyclooxygenase-2 (COX-2) mRNA (E) and interleukin-6 (IL-6) mRNA (F) versus HKG; n = 4–5 independent cell batches (Student’s *t*-test). *P<0.05, **P<0.01, ***P<0.001.

TNFα had marked effects on TET mRNA expression in endothelial cells and induced a decrease in TET1 (Fig 5A), was largely without effect on TET2 (Fig 5B), but increased TET3 (Fig 5C). Because TET enzymes reduce 5mC levels, a decrease in TET1 expression can stabilize 5mC. Indeed, the downregulation of TET1 in endothelial cells elicited a decrease in ACE protein levels (Fig 5D). Consistent with a previous report that decreased TET1 activity attenuated 5hmC in cyclooxygenase 2 and interleukin 6 [25], the expression on both genes increased following stimulation with TNFα or siRNA directed against TET1 (Fig 5E&5F).

## Discussion

ACE inhibitors have potent clinical effects and anti-inflammatory effects on the vascular wall [26]. Indeed, while endothelial cell activation is generally associated with enhanced ACE expression it is not generally appreciated that cytokines, such as TNFα, actually decrease ACE expression [6]. The results of the present study revealed that TNFα elicited the downregulation of ACE mRNA and protein in human endothelial cells by a mechanism at least partially regulated by DNMT3a, DNMT3b and TET1, and linked to increased methylation of the ACE promoter and decreased binding of the transcription factor MAX.

Previous reports have implicated nuclear factor κB and the p38 MAPK signaling pathway, in the regulation of endothelial cell ACE expression by TNFα [6]. However, by closely studying the time course of the TNFα-induced decrease in ACE expression as well as its recovery over 72 hours, it was evident that neither p38 MAPK nor mRNA destabilization processes played a role in the phenomenon observed. Rather, epigenetic regulatory mechanisms determined ACE levels in endothelial cells. Methylated DNA immunoprecipitation experiments confirmed the TNFα-dependent increase in 5mC content within the ACE promoter.

CpG islands are CG-rich regions with a CG content over 50% and an expected to observed CG ratio over 60%, covering a distance of at least 200 bp [27,28], and are often enriched at promoter sites and 5’-untranslated regions [26,29]. In general, promoter hypermethylation results in condensed chromatin structures and hence reduced transcription rates [30], largely due to hindered transcription factor binding. In this study the transcription factor MAX was the main focus as the ACE promoter region contains a CpG island that harbors two MAX binding sites. The latter transcription factor functions as an adapter protein for MAX dimerization protein (MAD) family members and binds the E-box sequence CANNTG [31,32]. Given that the binding affinity of MAX to DNA is highly sensitive to alterations in 5mC [32], and some MAD proteins, e.g. MAD1 and MAX gene-associated protein, are known to be regulated by short term (<24 hours) TNFα stimulation in human endothelial cells [33–35], it was tempting to speculate that the methylation of CpG-sites in the ACE promoter directly affected MAX binding. This turned out to be the case, as ChIP experiments revealed a decrease in the binding of MAX to those portions of the ACE promoter in which DNA methylation increased in response to TNFα stimulation. Ours is not the first study to suggest a role for DNA methylation in the regulation of ACE expression as DNA hypomethylation in fetal murine brains exposed to protein malnutrition *in utero* was previously linked with increased ACE mRNA expression [36]. Also, low birth weight in humans was linked to a decrease in the ACE methylation levels coupled with higher ACE activity [13]. Indeed, a significant inverse correlation was evident between the degree of DNA methylation and both ACE activity and systolic blood pressure levels [13]. ACE promoter hypermethylation in patients with clinical depression (which has been associated with cardiovascular disease), on the other hand, was linked with decreased ACE mRNA levels [37]. Although the latter observation has recently been questioned, it now seems that ACE genetic variants (seven polymorphisms across the ACE gene were studied) influence methylation and that ACE methylation is inversely correlated with cortisol levels [38]. The DNMT family of proteins (DNMT1, DNMT3a and DNMT3b), catalyze the transfer of a methyl group to a CpG-site. While DNMT1 preferably binds hemi-methylated DNA, DNMT3a and DNMT3b generally bind to non-methylated DNA and lead to *de novo* DNA methylation (for review see [26]). The siRNA-mediated downregulation of DNMT3a and DNMT3b largely prevented the TNFα-induced decrease in ACE mRNA and protein, while DNMT1, which is mainly responsible for the maintenance of DNA methylation, failed to affect ACE levels. Methylated CpG islands are very stable epigenetic marks and can be counterbalanced by the TET enzymes so that a decrease of TET expression can stabilize 5mC levels. Not only were DNMT3a and DNMT3b implicated in the response to TNFα, but cell stimulation also elicited a decrease in TET1, indicating that ACE levels were regulated by the reciprocal regulation of DNMT3a/b and TET1. While detailed data is not available for endothelial cells, a direct link between cytokine treatment, decreased TET1 activity and the loss of 5hmC marks has been described for a subset of genes in human chondrocytes [25], including cyclooxygenase 2 and interleukin 6. Importantly, the expression of both of these proteins was also increased in human endothelial cells following TET1 downregulation.

Taken together, the present study highlights the role of DNA methylation in the regulation of ACE expression in human endothelial cells following inflammatory activation. Given the positive effects of ACE inhibitors in the prevention and treatment of cardiovascular disease, it seems counterintuitive that ACE expression decreases in endothelial cells activated by inflammatory stimuli. However, ACE downregulation may, at least initially, help to dampen the inflammatory response by attenuating the metabolism of vasoprotective/anti-inflammatory ACE substrates, such as bradykinin and N-acetyl–seryl-asparyl-lysyl-proline at the same time as preventing the generation of angiotensin II. However, a similar response in the bone marrow would be expected to increase the mobilization of bone marrow-derived hematopoietic progenitor cells [39], may elicit the opposite effect and accelerate vascular inflammation and cardiovascular disease.

While ACE expression and activity is concentrated in endothelial cells in the lung and kidney, it is important to note that the ACE protein can be detected in several different cell types. These include, vascular smooth muscle cells, adipose tissue, leukocytes, macrophages and monocytes [40] as well as in cells lining the endosteal bone [39], primitive human embryonic hemangioblasts [41] and hematopoietic stem cells in both fetal and adult hematopoietic tissues [42]. In many cases, particularly in monocyte-derived macrophages [43] and vascular smooth muscle cells [44], it seems that the degree of cell differentiation/dedifferentiation markedly affects ACE levels. Although monocytes express ACE, and ACE expression increases during differentiation to macrophages [43], the functional consequences of enzyme regulation are unclear. Even though murine macrophages (in contrast to human macrophages) express only low levels of ACE [45], studies with transgenic mice genetically engineered to express high levels of the enzyme in macrophages, revealed an exaggerated inflammatory response that can limit melanoma growth [46]. A recent study in which the N- and C-catalytic domains of ACE were deleted revealed that TNFα blockade reverted the phenotype of ACE overexpressing macrophages lacking the N-terminal catalytic site to that of wild-type macrophages [47]. Given that TNFα is important for the classical activation of monocytes it would be interesting to compare the role of methylation and MAX in regulating ACE expression in endothelial cells and monocytes. It is certainly tempting to speculate that the TNFα generated during the classical activation of monocyte-derived macrophages may act to curtail the inflammatory response the promote cell repolarization to the alternatively activated phenotype that is associated with the resolution of inflammation.

## Supporting information

S1 Fig Consequences of pharmacological manipulation on the TNFα-induced changes in ACE expression. (A) Effect of R-7050 (10 μmol/L) on the expression of ACE mRNA in human endothelial cells treated with solvent (Sol) or TNFα (10 ng/mL, 24 hours); n = 3 independent cell batches. (B) Effect of DRB (20 μg/mL) on the expression of ACE mRNA in human endothelial cells treated with solvent (Sol) or TNFα (10 ng/mL, eight hours); n = 3 independent cell batches. (C) Consequences of p38 MAPK inhibition using SB202190 (SB; 5 μmol/L) on ACE expression versus a triplet of housekeeping genes (HKG) in the presence of solvent or TNFα; n = 4 independent cell batches (two-way ANOVA/Bonferroni). ***P<0.001.
(TIF)

S2 Fig Organization of the ACE gene and MAX expression. (A) Scheme: The *ACE* gene (first 3 of 26 exons are indicated by black boxes) presence a CpG island (gray box), that spans from -612 bp to +605 bp relative to the TSS (+1). Additionally, the gene contains two MAX binding sites: one from -1336 bp to -1135 bp upstream of the TSS (“upstream-site”) and another one within the CpG island (from +27 bp to +318 bp; “CpG-site”). (B) MAX expression versus NMM in endothelial cells cultured in the presence of solvent (Sol) or TNFα (10 ng/mL, 24 hours); n = 6 independent cell batches (Student’s *t*-test).
(TIF)

S3 Fig Efficiency of DNMT downregulation in human endothelial cells. DNMT mRNA expression in endothelial cells, which were transfected with siRNAs directed against DNMT1, DNMT3a, DNMT3b or a scrambled (Scr) oligonucleotide as control; n = 4–8 independent cell batches (Student’s t-test), ***P<0.001.
(TIF)

S1 File PDF with the proteomics data of the differentially expressed proteins. The criteria are those mentioned in Materials and methods.
(PDF)

S2 File Excel file with the data used for quantification of mRNA and protein. The criteria are those mentioned in Materials and methods.
(XLSX)
