# Supplementary figures and images for: Epigenetic control of the angiotensin-converting enzyme in endothelial cells during inflammation
Source: PLoS One. 2019 May 1;14(5):e0216218. doi: 10.1371/journal.pone.0216218 (PMC6494048; doi:10.1371/journal.pone.0216218)

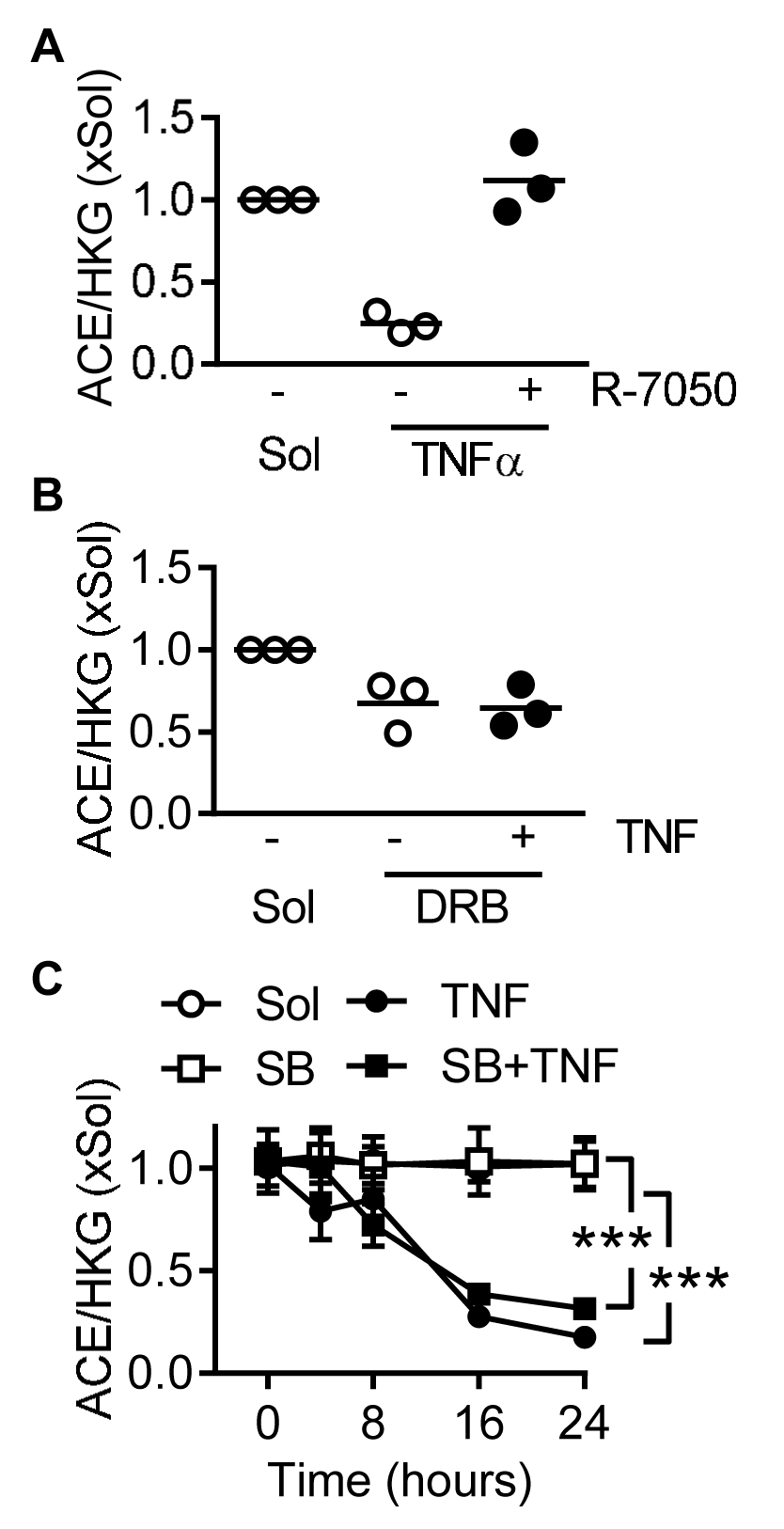

Supplement: S1 Fig — (A) Effect of R-7050 (10 μmol/L) on the expression of ACE mRNA in human endothelial cells treated with solvent (Sol) or TNFα (10 ng/mL, 24 hours); n = 3 independent cell batches. (B) Effect of DRB (20 μg/mL) on the expression of ACE mRNA in human endothelial cells treated with solvent (Sol) or TNFα (10 ng/mL, eight hours); n = 3 independent cell batches. (C) Consequences of p38 MAPK inhibition using SB202190 (SB; 5 μmol/L) on ACE expression versus a triplet of housekeeping genes (HKG) in the presence of solvent or TNFα; n = 4 independent cell batches (two-way ANOVA/Bonferroni). ***P<0.001. (TIF) [file pone.0216218.s001.tif]

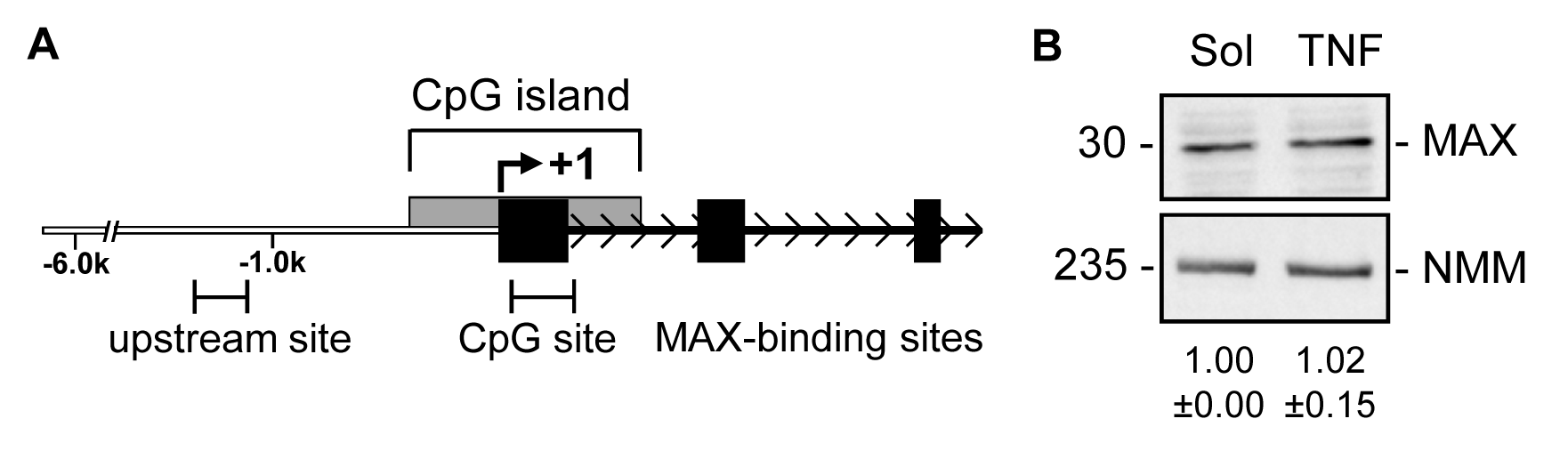

Supplement: S2 Fig — (A) Scheme: The ACE gene (first 3 of 26 exons are indicated by black boxes) presence a CpG island (gray box), that spans from -612 bp to +605 bp relative to the TSS (+1). Additionally, the gene contains two MAX binding sites: one from -1336 bp to -1135 bp upstream of the TSS (“upstream-site”) and another one within the CpG island (from +27 bp to +318 bp; “CpG-site”). (B) MAX expression versus NMM in endothelial cells cultured in the presence of solvent (Sol) or TNFα (10 ng/mL, 24 hours); n = 6 independent cell batches (Student’s t-test). (TIF) [file pone.0216218.s002.tif]

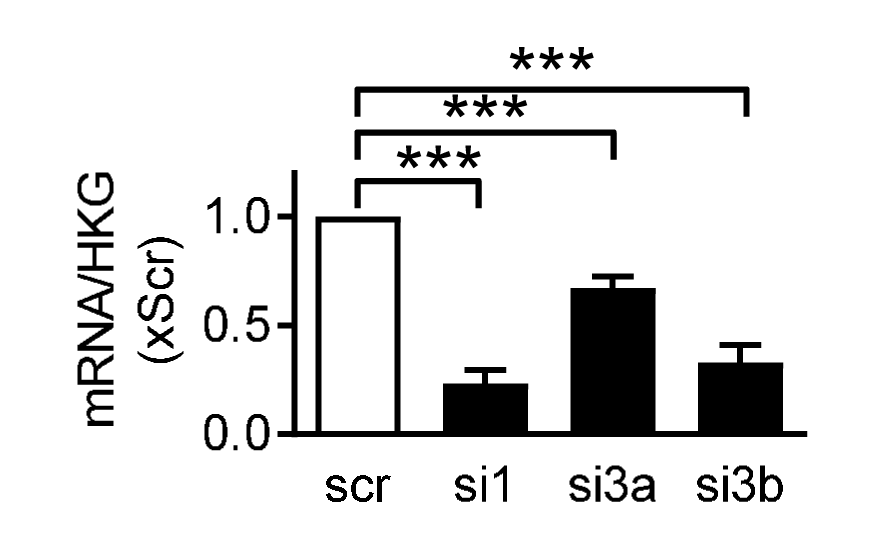

Supplement: S3 Fig — DNMT mRNA expression in endothelial cells, which were transfected with siRNAs directed against DNMT1, DNMT3a, DNMT3b or a scrambled (Scr) oligonucleotide as control; n = 4–8 independent cell batches (Student’s t-test), ***P<0.001. (TIF) [file pone.0216218.s003.tif]

Figure 1

**B**

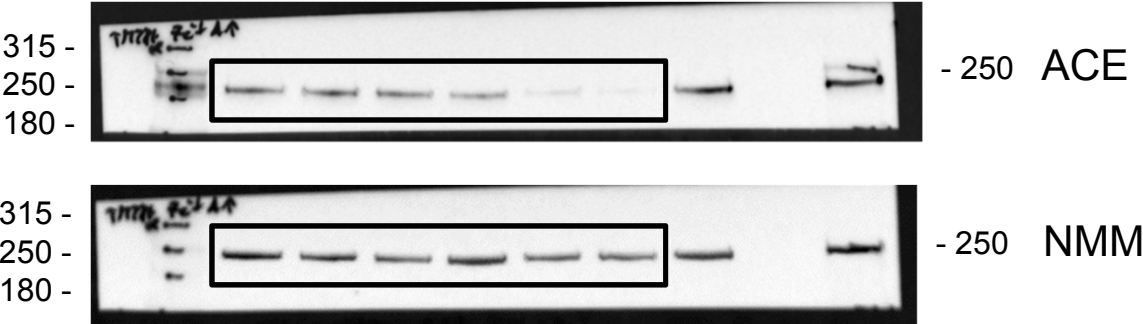

**D**

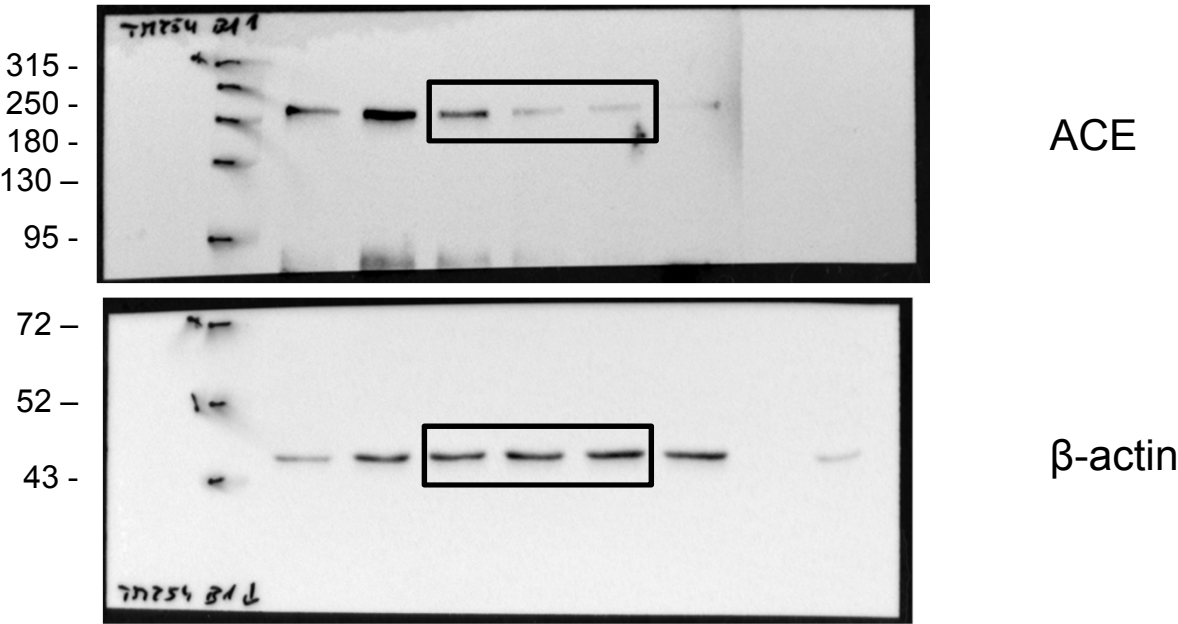

Figure 2

**B**

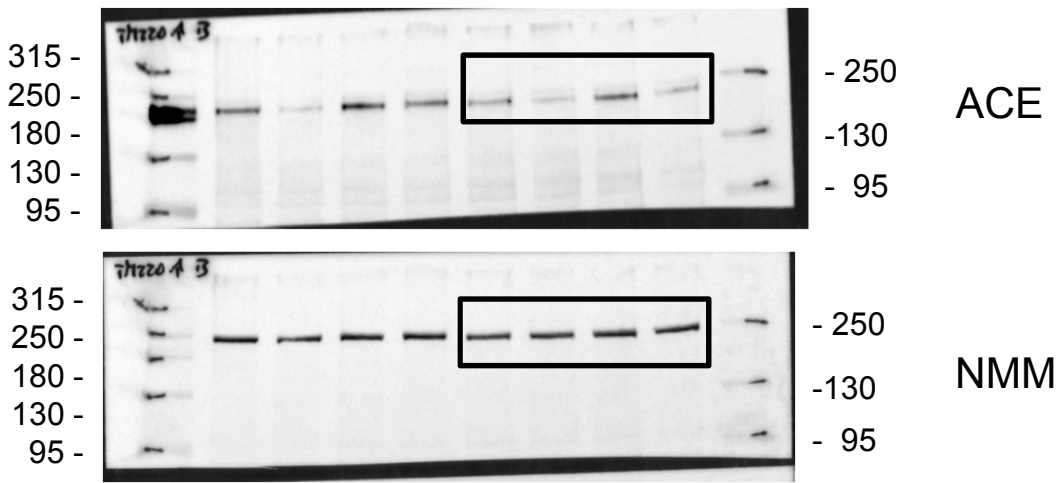

### Figure 3

# E

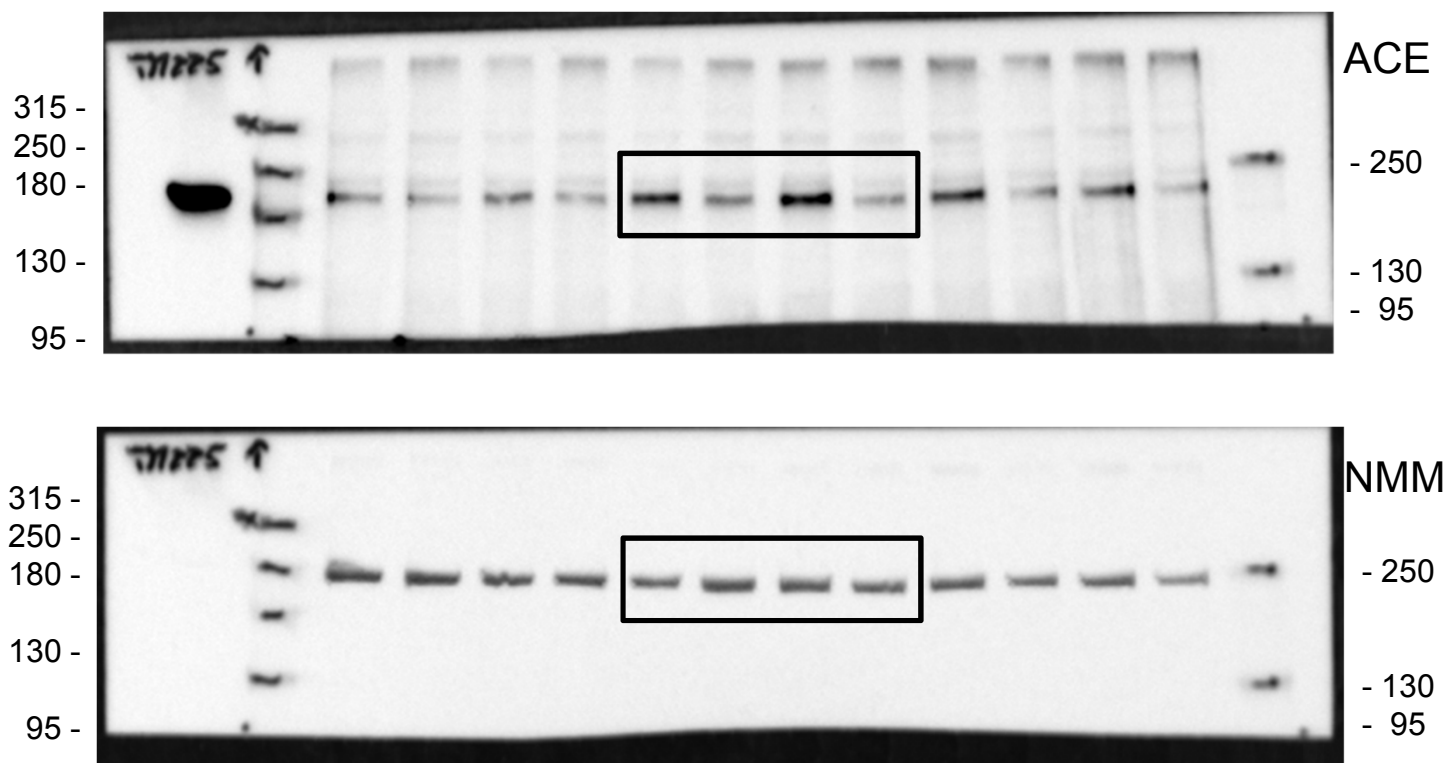

Figure 4

C

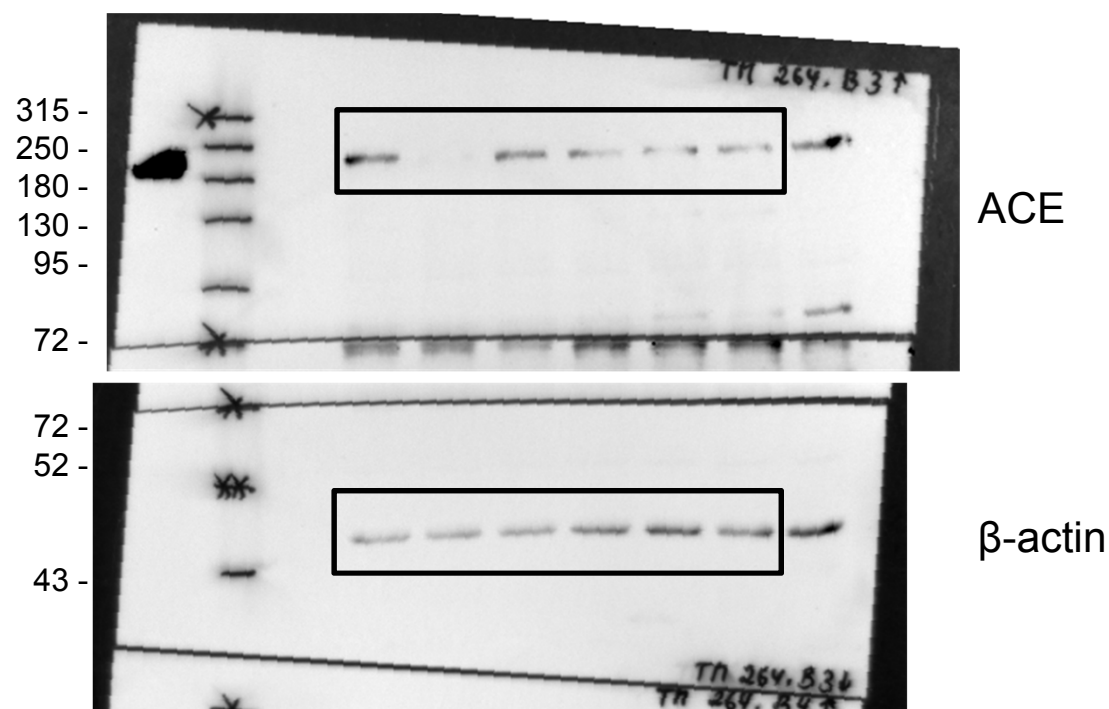

Figure 5

D

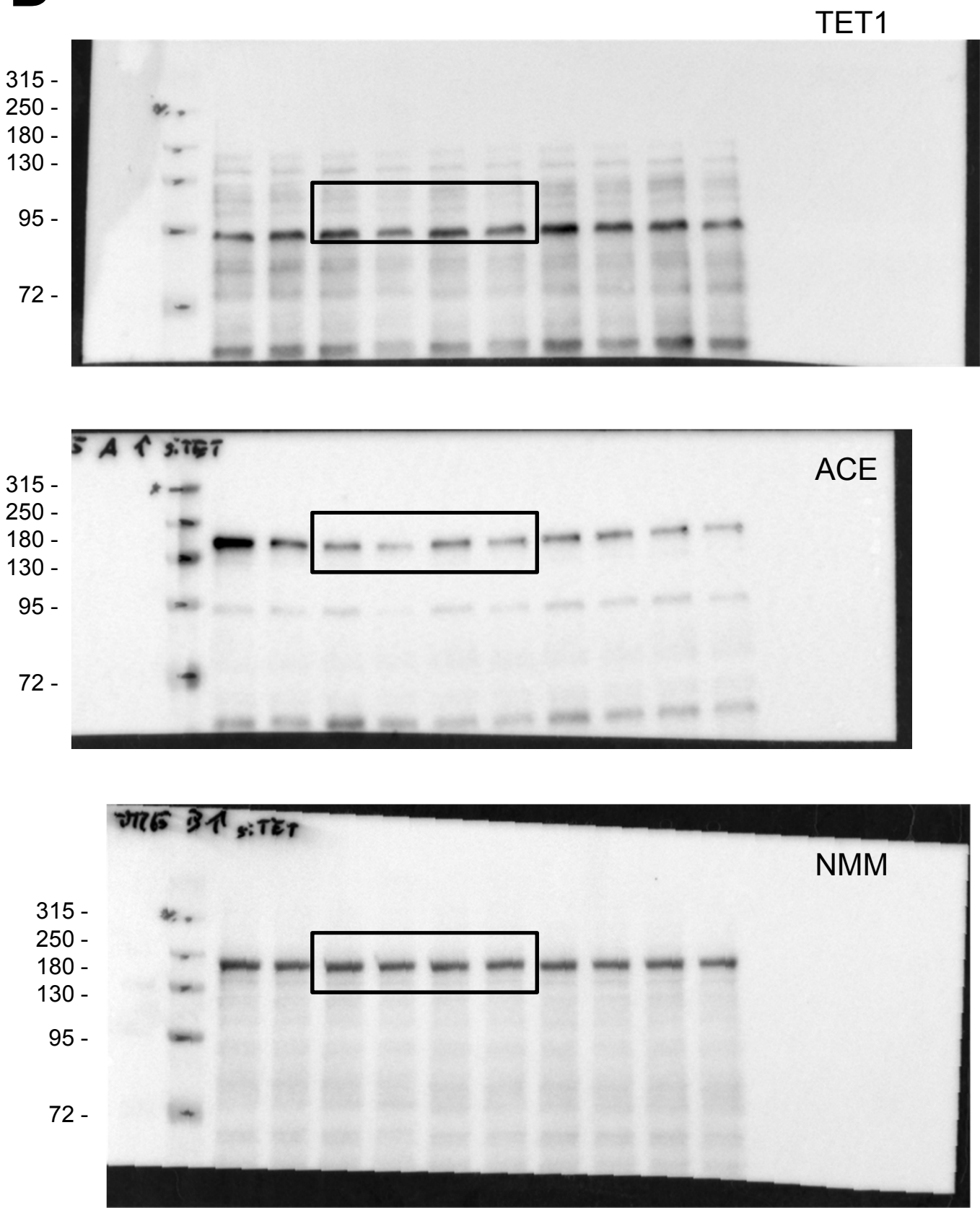

# Supplement Figure S2

**B**

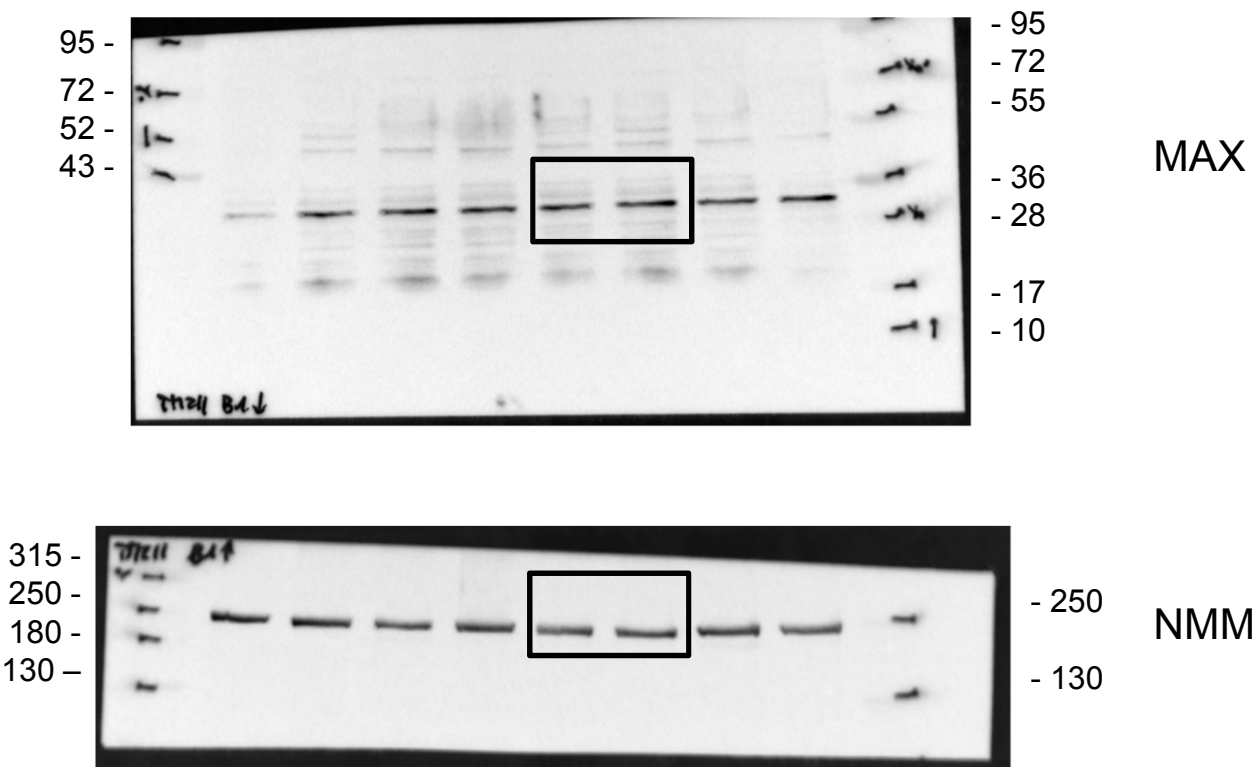

Supplement: S1 File — The criteria are those mentioned in Materials and methods. (PDF) [file pone.0216218.s004.pdf]
